# When Do Physicians Trust AI? The Role of Clinical Context in Decision Support: SMART vignette study

**DOI:** 10.21203/rs.3.rs-10760935/v1

**Published:** 2026-09-10

**Authors:** Bohye Kim, Katie Ryan, Max Kasun, Jane Paik Kim

**Affiliations:** Stanford University School of Medicine; Stanford University School of Medicine; Stanford University School of Medicine; Stanford University School of Medicine

**Keywords:** SMART, AI-CDSS, information, prior exposure to AI, clinical decision-making

## Abstract

**Background:**

Physicians’ confidence in clinical AI tools and perceptions of their effectiveness can influence the adoption of these tools in practice, which in turn, could affect the quality of patient care. However, the factors shaping these perceptions remain poorly understood.

**Methods:**

We surveyed U.S. family medicine and internal medicine physicians (n = 420) using a sequential multiple-assignment randomized trial (SMART) vignette design involving primary care scenarios with an AI-enabled clinical decision support system (AI-CDSS). We varied three vignette characteristics, each at two levels: clinical decision types (prescription or referral), amount of information provided about the AI-CDS tool (more or less), and the tool’s recommendations (treat or do not treat). We used regression models to assess whether clinical decision type or amount of information about the AI-CDSS affected physicians’ confidence and their perceptions of AI-CDSS’s effectiveness, use of AI in clinical practice, and clinical decisions. We also examined associations between physicians’ prior AI exposure and these perceptions.

**Results:**

Physicians’ prior exposure to AI was positively associated with their confidence in their decisions (β = 0.274, adjusted p = 0.042) and perceptions of effectiveness of the AI-CDSS (β = 0.350, adjusted p = 0.006). Neither clinical decision type nor amount of information significantly affected perceived confidence or effectiveness. Exploratory analyses revealed that physicians assigned to a prescription scenario reported lower trust in the AI-CDSS (β=−0.211, p = 0.027), fewer benefits of using it (β=−0.179, p = 0.042), and greater reliance on their own judgment (β = 0.154, p = 0.029) versus those assigned to a psychiatric referral scenario. Prior AI exposure was also associated with greater agreement that the AI result contributed to the final decision (β = 0.305, p = 0.010).

**Conclusions:**

Our findings suggest that prior knowledge of and exposure to AI may enhance physicians’ confidence in their clinical decisions and their perceptions of the effectiveness of AI-CDS tools, underscoring the importance of predeployment assessment of human factors in clinical AI implementation. Training in AI use and AI-assisted decision-making should be integrated into physicians’ education, while factors influencing physicians’ confidence merit continued examination.

## 1. BACKGROUNDS

Artificial intelligence-driven clinical decision support systems (AI-CDSS) are being increasingly integrated into healthcare workflows. Yet the promise they hold for clinical care depends fundamentally on clinician oversight and judgment, informed by education, training, and expertise, and is constrained by whether these tools can be reliably, empirically, and carefully embedded into clinical ecosystems. Effective, ethically robust integration of AI-CDSS therefore requires our enhanced understanding of how clinicians critically evaluate, interpret, and reach decisions in concert with AI recommendations, and understanding how algorithmic outputs may be assimilated with independent clinical judgment drawn from clinicians’ backgrounds and other relevant information outside the scope of a deployed AI-CDSS.

Despite surging regulatory approvals for AI-enabled clinical tools and enthusiasm for AI-enabled clinical tools, less than 2% of AI models, to date, have advanced beyond a prototyping phase into clinical use.(1) A limited body of ethics literature has identified successful clinical integration of novel AI-CDS tools is heavily shaped by clinicians’ trust in AI-CDS tools, perceived adverse disruptions of clinical workflows caused by premature integration, and other challenges that plausibly could disrupt or undermine clinicians’ therapeutic relationships with patients and ultimately adversely impact AI-CDSS-assisted care. (2–4) For example, a brief systematic review and meta-analysis of studies of CDSS-driven “alert fatigue” (i.e. overwhelming volume and/or intensity of devices’ sensory cues such that they distract and impair decision making) found that physicians overrode upwards of 90% of alerts, with many overrides seemingly driven by cognitive burden among competing obligations and tasks rather than governed predominantly by intent and clinical reasoning.(5) On the other hand, a prospective study regarding an LLM (Large Language Model)-based AI tool generating hospital course summaries suggested that physician burnout scores significantly decreased after using the AI tool although the time savings were modest.(6) Such gaps between algorithmic performance and clinical effectiveness highlight the need for more empirical assessment of clinician perceptions in the translation of AI-CDS tools into clinical settings, both to improve patient care and to safeguard patients from premature implementation of tools that could introduce unmitigated and undue risks or lead to outright harm.(7, 8)

Further complicating the successful uptake of AI-CDSS in medicine are state- and context-dependent characteristics of human clinical judgment, including risk perception or how information is framed.(9, 10) Empirical work has revealed that the perceived risk of the clinical decision at hand;(11) quality of explanation about the AI-CDSS mechanism (e.g., accuracy, semantic transparency); and a clinician’s own training, prior exposure, and familiarity with the underlying tool were important factors that may influence physicians’ perceptions of AI integration.(10) In a recent article, Gilbert et al. discussed the need for transparent and standardized information about health AI tools as well as layered information tailored to different stakeholders and needs within the healthcare system.(12) This emphasizes that how much information is disclosed regarding AI-CDSS may influence physicians’ trust in the tools and effectiveness. In concert, these factors may shape whether a given AI recommendation is followed, scrutinized, or ignored, holding consequences for diagnostic accuracy and health care systems’ ability to adequately allocate human attention and other resources.(13)

Despite the urgency to better understand AI-CDS tools’ influences on clinician judgment, prior work has largely examined factors in isolation without considering their relationships to one another, their relative importance, or how they may interact in real-world clinical contexts. As AI-CDS tools become more prevalent in primary care where about 1 in 6 patient visits are for serious mental health concerns,(14) increasing understanding of the quality of clinical judgments made alongside AI-CDS tools is urgent. A better understanding of which systemic factors influence physician trust, confidence, and reliance on AI tools may help in advancing implementation strategies that optimize clinical benefit and safeguard against introducing new sources of error.

To this end, we used a novel survey design method called SMART vignettes, which allowed us to examine physician responses to different clinical scenarios based on prior responses and allocation. We sought to understand their confidence and perceived quality of care in response to (1) the type of clinical decision and its risk profile, (2) the amount of information disclosed about the AI-CDSS, and (3) physician prior exposure to AI, conditional on different clinical contexts and their responses to other factors.

## 2. METHODS

### 2.1 Participants

Participants in this online survey study were active U.S. physicians (M.D.s or D.O.s) with subspecialties in Family Medicine or Internal Medicine (n = 420). We contracted with Medical Marketing Services, Inc. (MMS) to recruit a sample of physicians from the American Medical Association (AMA) Physician Professional Data file.(15) Based on the results of previous survey studies utilizing a similar recruitment strategy, a response rate of approximately 5% was anticipated.(16) As our target sample size was 420 based on a power calculation, we randomly sampled 10,000 physicians from the most recent AMA Physician Professional Data file who met the inclusion criteria of being located in the United States and having a listed subspecialty of Family Medicine or Internal Medicine. Between May and December 2023, physicians were contacted via email and physical mailing addresses with invitations containing a brief description of the study and a weblink to participate. The target sample size of 420 was reached on 23 January 2024, at which point the survey was closed. Physicians who completed this 10–15-minute survey received a $10 Amazon.com gift card upon completion.

### 2.2 Study Design

#### SMART Vignette Survey.

This study utilized a SMART vignette design, an online adaptation of the sequential multiple assignment randomization trial (SMART) design for vignette research.(17, 18) The SMART vignette approach introduces distinctive characteristics not previously employed in vignette methodology, namely sequential randomization combined with adaptive assignment. Conventional vignette research typically presents respondents with a single narrative scenario containing all variations of experimental factors alongside a complete question set. In contrast, SMART surveys decompose vignettes into sequential micro-scenarios (“subvignettes”) where individual micro-scenarios capture specific dimensions (i.e., of decision types or the amount of information) that are systematically varied across the respondent sample. The subvignettes are presented sequentially, each paired with a brief question set. Survey respondents receive subvignettes through probabilistic assignment designed to achieve balanced data collection across subvignettes.(18)

The SMART survey was administered using the platform developed by Kim and Yang (2024)(17) and hosted on “smartvignettes.stanford.edu”. The vignette survey featured three time points at which participants were randomized to subvignettes reflecting different clinical decision types (prescription or referral), different amounts of information on the AI-CDS tool (more or less), and different recommendations of the tool’s output (treat or do not treat). Participants were presented with 21 total questions across three different time points. Questions primarily consisted of 5-point Likert scales, with the exception of two that allowed for binary responses and another that allowed a free response. A primary question was chosen for each time point to be used for adaptive allocation with p = 2/3 (i.e., biased coin design).(18) The exact subvignettes and their accompanying question sets can be found in the Supplement (Supplemental Table 1). Figure 1 depicts the setup of our SMART survey.

#### Vignette Content.

Vignette narratives were constructed by the research team to convey different levels of the variables being tested (Table 1). This approach enabled us to control the elements in the vignette and focus on the impact of the factors of interest (i.e., decision type, information framing) on physician perceptions without limiting our study to a specific AI model or version.

### 2.3 Study Aims and Hypotheses

The primary aims of this study were to test the impact of the type of clinical decision assisted by the AI- CDSS and the amount of provided information about the AI-CDSS on physicians’ ratings of confidence in and effectiveness of the AI-CDSS. We also aimed to test whether prior exposure to AI was associated with ratings of confidence in and effectiveness of the AI-CDSS, and whether prior exposure moderated the effect of the amount of information on confidence and effectiveness.

We hypothesized the following: (1) A clinical decision involving higher risk, compared to lower risk, would negatively influence physicians’ ratings of confidence in the AI-CDSS and effectiveness of the AI-CDSS, while accounting for the amount of information provided on the AI-CDSS and physicians’ prior exposure;(2) more information disclosed about the AI-CDSS would positively impact physicians’ ratings of confidence in the AI-CDSS and effectiveness of the AI-CDSS, while accounting for the clinical decision type and physicians’ prior exposure; and (3) exposure to AI would be positively associated with physicians’ ratings of confidence in the AI-CDSS and effectiveness of the AI-CDSS, and be a moderator for the effects of additional information (i.e., more information disclosed about the AI would positively impact ratings of confidence and effectiveness for physicians with prior exposure to AI, but would have a negative to neutral impact for physicians without exposure).

As an exploratory analysis, we examined the impact of the decision type and the amount of information on physicians’ perceptions of AI use in practice and clinical decisions. We also explored how physicians’ prior exposure to AI moderated these impacts, as well as associations between physicians’ exposure to AI and physicians’ perceptions.

### 2.4 Measures

#### Outcomes.

The primary outcomes were self-reported responses regarding confidence in the AI-CDSS in helping the physician make the best treatment decision for their patient (i.e., “I am confident in this decision”; “The MedAssist result improved the confidence I have in my final decision”), as well as the perceived effectiveness of the AI-CDSS (i.e., “Overall, MedAssist improved the care that I was able to provide to the patient”).

Exploratory outcomes were physicians’ perceptions of AI use in practice (asked at Timepoint 2) and clinical decisions (asked at Timepoint 3). The ratings were on a 5-point Likert scale (1 - Strongly disagree to 5 - Strongly agree; or 1 - I relied entirely on MedAssist to 5 - I relied entirely on my own examination), except two binary exploratory outcomes (1 - Yes, 0 - No): “If given the option, I would choose to use MedAssist in my practice” and “Based on your own examination and the MedAssist result, would you prescribe an SSRI to Jess / refer Jess for a formal ADHD assessment?”

#### Predictors.

Clinical decision type (prescribing = 1, referral = 0; representing higher vs. lower risk, respectively) and the amount of information about the AI (more = 1, less = 0; representing more vs. less information disclosed about the AI-CDSS, respectively) were used as predictors.

Exposure to AI, a baseline characteristic of physicians, was included as a predictor and interaction term. Physicians were classified as having prior exposure to AI if any of the following were true: a response of “yes” to “In your research, have you ever made use of artificial intelligence or machine learning?” or “In your clinical practice, have you ever made use of artificial intelligence or machine learning?”; or a response of 4 (“Quite familiar. I could partially explain what it is and how it works”) or 5 (“Extremely familiar. I could explain in detail what it is and how it works”) to the question “How would you describe your overall familiarity with artificial intelligence and machine learning?”

### 2.5 Statistical Analysis

To test Hypotheses 1 and 2, we performed linear regression with each primary outcome as the dependent variable, and clinical decision type, the amount of information, and exposure to AI as independent variables. For Hypothesis 3, we performed linear regression using each primary outcome regressed on the amount of information, exposure to AI, and the interaction between the amount of information and exposure to AI. In the analysis for each hypothesis, we applied one-sided tests at a significance level of 0.05, along with the Benjamini&Hochberg correction (BH procedure) for multiple testing across the outcomes.

For our exploratory aims, we repeated the analytic approach used for Hypotheses 1, 2, and 3 (i.e., using the same independent variables) with two-sided tests at a significance level of 0.05 across the exploratory outcomes. For two binary outcomes, logistic regressions were used as appropriate.

All statistical analyses were conducted with R software (R V.4.3.1, GNU project).

## 3. RESULTS

Participant characteristics are presented in Table 2.

### 3.1 Primary Analyses

#### Impact of the clinical decision type, the amount of information, and AI exposure on physicians’ ratings of confidence and effectiveness.

The clinical decision type and the amount of information did not impact physicians’ ratings of confidence in or effectiveness of the AI-CDSS (Table 3). Physicians’ prior exposure to AI had positive associations with their confidence (“The MedAssist result improved the confidence I have in my final decision”: β = 0.274, adjusted p = 0.021) and ratings of the AI’s effectiveness (“Overall, MedAssist improved the care that I was able to provide to the patient”: β = 0.350, adjusted p = 0.003). In fact, ratings of trust, comfort, risk and benefit, and willingness to use the AI-CDSS in their practice were not associated with prior exposure.

#### Moderation effects of AI exposure on the impact of the amount of information on physicians’ ratings of confidence and effectiveness.

Physicians’ prior exposure to AI did not moderate the effects of the amount of information on their confidence (“I am confident in this decision”: β = 0.045, p = 0.393, adjusted p = 0.787; “The MedAssist result improved the confidence I have in my final decision”: β=−0.068, p = 1.000, adjusted p = 0.787) or ratings of the AI’s effectiveness (“Overall, MedAssist improved the care that I was able to provide to the patient”: β=−0.225, p = 1.000, adjusted p = 0.787).

### 3.2 Exploratory Analyses

Figure 2 illustrates the regression coefficients for clinical decision type, the amount of information regarding the AI-CDSS, and physicians’ prior exposure to AI on their perceptions regarding AI use in practice (i.e., the questions asked at Timepoint 2) and their anticipated clinical decisions involving the AI (i.e., the questions asked at Timepoint 3).

#### Impact of the clinical decision type on outcomes.

Physicians randomized to the prescription scenario expressed less agreement with trusting MedAssist to provide consistent recommendations for all patients (β=−0.211, p = 0.027), and expressed less agreement that the benefits of using MedAssist would outweigh any risks (β=−0.179, p = 0.042), than those randomized to the referral scenario. Physicians randomized to the prescription scenario expressed greater agreement with relying on their own examination (versus the MedAssist recommendation) than those randomized to the referral scenario (β = 0.154, p = 0.029; see Supplemental Table 2).

#### Impact of the amount of information on outcomes.

Physician’s perceptions of using AI-CDSS in practice or making clinical decisions did not differ by the amount of information provided about the AI-CDSS (see Supplemental Table 2).

#### Association of AI exposure with outcomes.

Physicians’ prior exposure to AI was associated with higher levels of physician agreement that the AI result contributed to their final clinical decision (β = 0.305, p = 0.010). Physicians’ prior exposure to AI did not moderate the effects that the amount of information had on any of the exploratory outcomes regarding physicians’ perceptions of AI use in practice or clinical decisions (see Supplemental Table 3).

## 4. DISCUSSION

The accelerating integration of AI-based decision-support tools into primary care necessitates a thorough understanding of physician attitudes towards these technologies, as physicians’ acceptance or, conversely, skepticism of AI tools can significantly impact their effectiveness and implementation. The gap between algorithm development and meaningful real-world clinical impact continues to limit the benefit of clinical AI tools, with implementation science pointing to human factors as a primary driver of this translation gap.(3, 4, 7, 19) Using a SMART vignette approach,(17) our study sought to address this gap by drawing inferences about the impact of a series of factors including clinical decision type and amount of information about the AI-CDSS on physician attitudes and clinical decision-making.

Contrary to our initial hypothesis, the amount of information provided about the AI-CDSS showed no significant effect on physician confidence or perspectives on effectiveness of the AI-CDSS. In a prior study by our team with patients, we found that a slightly more detailed description about an AI-CDSS used in their hypothetical care improved their comfort and confidence in the usage of the tool, as well as their perception of the physician’s final decision.(20) It also seems plausible, given the widely reported findings on physicians who are often overburdened and report feeling overwhelmed with the volume of alerts, in addition to electronic patient information that they interact with,(5) that additional information is not immediately impactful.

Physicians in our study appear to have differentiated between two types of risk, with those assigned to the “prescribing” scenario expressing less trust and confidence in and less reliance on the presented AI-CDSS when compared to those assigned to the “referring” scenario. These results corroborate the findings of prior studies, which have demonstrated that, in higher risk clinical scenarios, physicians report more skepticism regarding the use of AI-CDSS and greater reliance on their own clinical judgment versus the recommendation of an AI-CDSS.(8, 10) As AI is increasingly integrated into clinical care, it is important to recognize that even minor differences in the clinical scenario may influence physicians’ trust in and use of these systems. Given the stakes of clinical decisions, the integration of AI into healthcare necessitates a clearer understanding of which tasks physicians would be willing to defer to AI, versus those in which they prefer to be the primary decision-maker.

Recent studies have documented that physicians with greater AI familiarity express greater enthusiasm toward AI integration in medicine.(21) Our work suggests that physicians with prior exposure in our study rated *their own work performance and clinical decision-making involving the AI-CDSS* more positively (i.e., MedAssist “improved the confidence I have,” “improved the care that I was able to provide,” and “contributed to my decision”), but they did not rate *the AI-CDSS itself* more positively. This implies that physicians’ prior exposure to AI tools may be an important human factor in the clinically effective deployment of AI-CDS tools in healthcare settings.

### Limitations.

Limitations of the current study include the use of vignette-based methods and self-reported outcomes. While the outcomes were not observed in a real-time healthcare setting, we argue that this type of work fits squarely within pre-deployment safety assessments in higher-stakes domains where the potential impacts of human-in-the-loop systems should be thoroughly understood before deployment. While vignette-based predeployment assessment represents a rigorous and ethically important methodology for characterizing human factors before clinical AI tools are introduced into practice, studies of embedded learning healthcare settings linking decisions to patient outcomes would be the logical next step in determining the impact of physician decision-making with embedded AI. Our low response rate may be another limitation, although our observed rates were consistent with other studies utilizing similar recruitment strategies using the AMA Physician Professional Data file.(15)

## 5. CONCLUSIONS

Our finding that physicians’ prior exposure to AI was associated with greater confidence in their decisions and more favorable perceptions of AI-CDSS effectiveness underscores the importance of AI training for physicians. Therefore, training future physicians, who will likely use AI in clinical practice, should emphasize decision-making when weighing AI-generated input alongside patient context and clinical judgement rather than deferring to algorithmic recommendations. Physicians’ trust in AI will play a critical role in determining the extent of its adoption in clinical practice. Because inappropriate reliance on AI can pose substantial risks to patients, it is important to anticipate when such reliance may occur and develop safeguards that promote appropriately calibrated trust. Further work is needed to develop safeguards that align physicians’ confidence in AI with a given model’s reliability and limitations. Such safeguards may include audits or physician feedback mechanisms.

Understanding factors that influence physicians’ decisions, perceptions, or acceptance of AI may also inform the design of AI user interfaces, including whether to highlight data quality, model uncertainty, or the basis for an AI’s recommendation. Although we found that providing additional information did not significantly impact physicians’ confidence in their decisions or perceptions of AI-CDSS effectiveness, recent interest in information labels for AI tools suggests that the presentation of AI tools may itself shape its use.(12) Future studies examining how potential user interface or presentation factors, such as visual elements, explanatory styles, and data format, affect physicians’ perceptions, use, or adoption of AI will be valuable.

## Supplementary Material

This is a list of supplementary files associated with this preprint. Click to download.

- SmartVignettesAICDSSsupplementary.docx

## Figures and Tables

**Figure 1:**
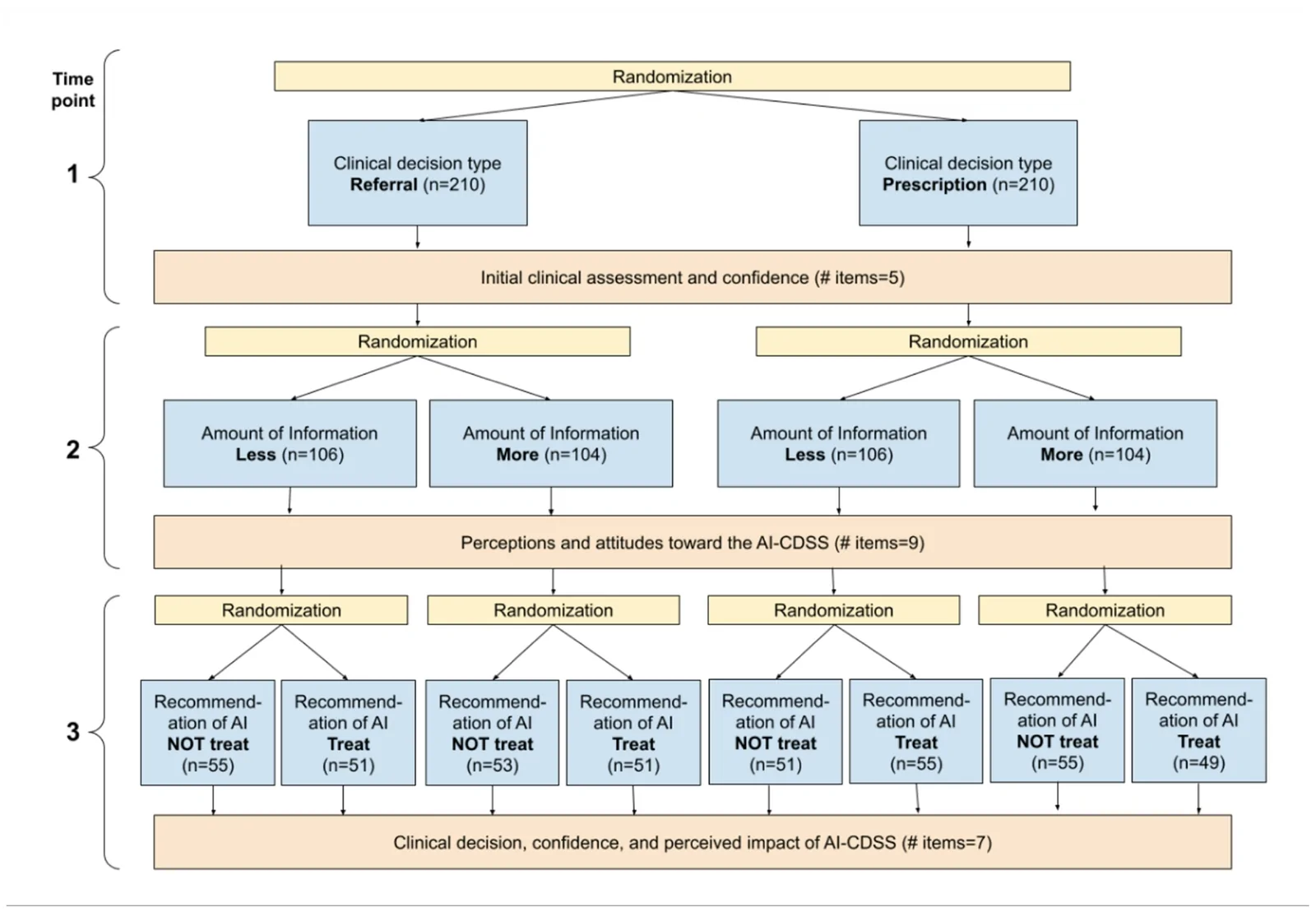
Setup of the SMART Survey (adapted from Kim et al., 2024). Participants were sequentially randomized at three time points to subvignettes reflecting different clinical decision types (prescription or referral), different amounts of information about the AI tool (more detailed or less detailed), and different AI output recommendations (treat or do not treat). Adaptive allocation used a biased coin design (p=2/3) at each time point.

**Figure 2:**
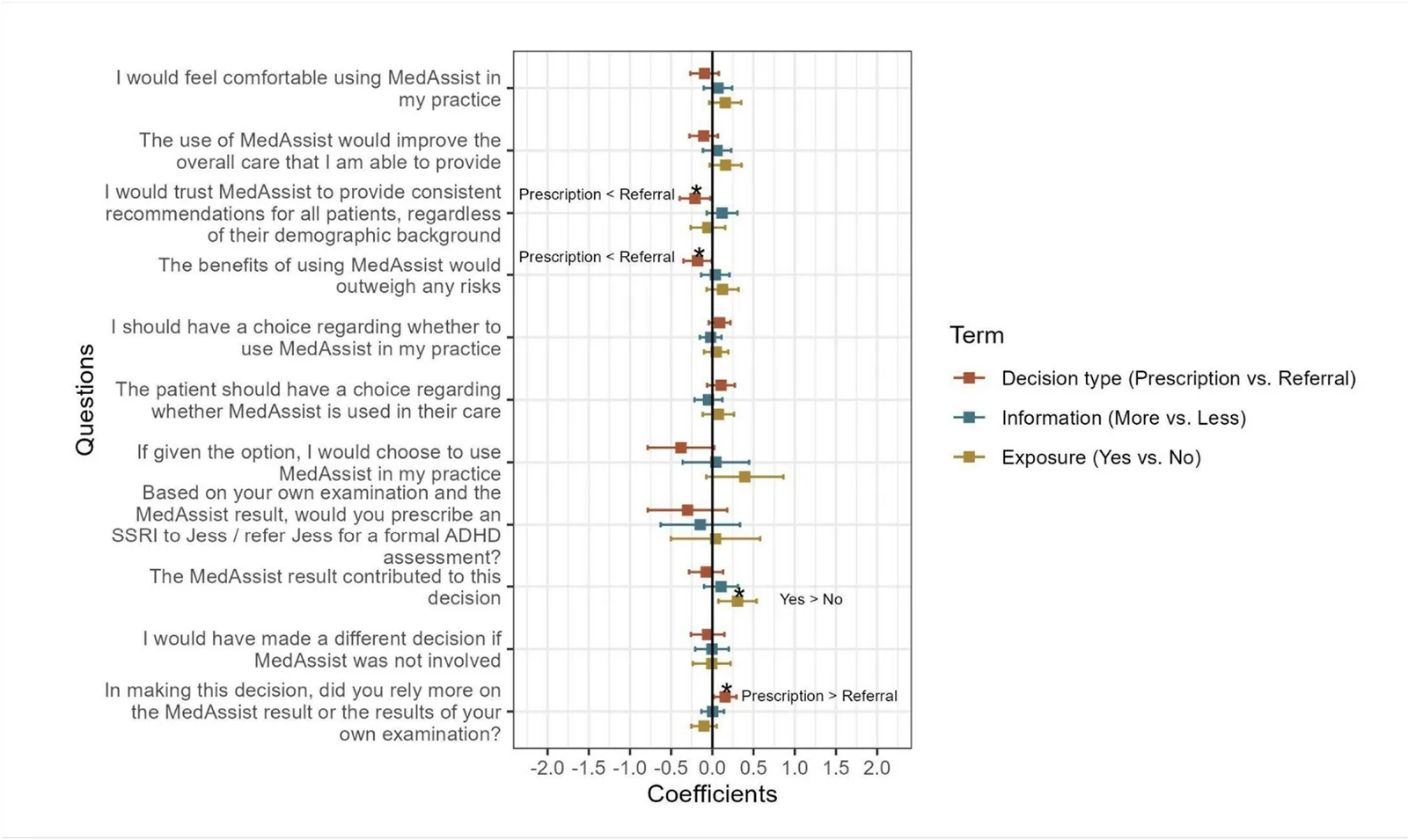
Coefficients of Clinical Decision Type, Amount of Information on AI, and Physician Exposure to AI on Physicians’ Perceptions of AI Use in Practice and Clinical Decisions. Squares represent point estimates for the regression coefficients; error bars indicate 95% confidence intervals. Statistically significant coefficients (error bars not crossing zero) are marked with an asterisk. Coefficients greater than 0 indicate that physicians randomized to prescription, physicians randomized to more information, or physicians with prior AI exposure show greater agreement with the statement compared to their respective counterparts; coefficients less than 0 indicate the reverse.

**Table 1:** Overview of vignette characteristics.

Clinical decision type^a^	A 33-year old female presents with symptoms of ADHD which may require a referral to a specialist.	A 33-year old female presents with symptoms of depression which may require prescription of an SSRI.
**Amount of information about the AI** ^a^	**5 sentences**, including details about: - The hospital’s use of the AI - How to use the AI - The patient records that the AI uses to determine a score	**10 sentences**, including details about: - The hospital’s use of the AI - How to use the AI - The patient records that the AI uses to determine a score **- Where the AI was developed and trained** **- The data used to train the AI** **- The AUC value of the AI**
**AI recommendation** ^a^	The AI output states that the patient is in the 52nd percentile in terms of compatibility, and therefore **does not recommend treatment**.	The AI output states that the patient is in the 95th percentile in terms of compatibility, and therefore **recommends treatment**.

a. Each participant was assigned one characteristic from each row (see Fig. 1). The full text of the vignettes can be found in the supplement.

**Table 2:** Participant Characteristics (N = 420)

	Overall
	(N = 420)
**Age**, years	
Mean (SD)	49.6 (12.5)
Median [Min, Max]	49.0 [28.0, 75.0]
Missing	3 (0.7%)
**Sex**	
Female	178 (42.4%)
Male	241 (57.4%)
Decline to state	1 (0.2%)
**Gender** ^a^	
Woman	180 (42.9%)
Man	238 (56.7%)
Nonbinary or Genderqueer	1 (0.2%)
Decline to state	1 (0.2%)
**Ethnicity**	
Not Hispanic or Latino	390 (92.9%)
Hispanic or Latino	22 (5.2%)
Decline to state	8 (1.9%)
**Race**	
American Indian/Alaska Native	4 (1.0%)
Asian	103 (24.5%)
Black or African American	17 (4.0%)
Native Hawaiian or Other Pacific Islander	1 (0.2%)
White	256 (61.0%)
Multiracial	16 (3.8%)
Other	9 (2.1%)
Decline to state	14 (3.3%)
**Degree**	
M.D.	367 (87.4%)
M.D./ Ph.D.	7 (1.7%)
D.O.	45 (10.7%)
D.O./ Ph.D.	1 (0.2%)
**Training** ^a^	
Medical school - USA	335 (79.8%)
Medical school - Abroad	80 (19.0%)
Post-graduate - USA	59 (14.0%)
Post-graduate - Abroad	10 (2.4%)
Other	1 (0.2%)
Decline to state	1 (0.2%)
**Practice years**	
Mean (SD)	18.3 (12.0)
Median [Q1, Q3]	17.0 [8.00, 26.0]
Missing	1 (0.2%)
**Primary subspecialty**	
Family medicine	216 (51.4%)
Internal medicine	204 (48.6%)
**Practice Region**	
Northeast	94 (22.4%)
Southeast	94 (22.4%)
Midwest	97 (23.1%)
Southwest	33 (7.9%)
West	95 (22.6%)
Decline to state	7 (1.7%)
**Formal training/education in computer science**	
Yes	81 (19.3%)
No	339 (80.7%)
**Type of computer science training/education** ^a^	
High school course(s)	34 (8.1%)
Undergraduate course(s)	53 (12.6%)
Graduate-level course(s)	12 (2.9%)
CS/Coding bootcamp	6 (1.4%)
Professional development course(s) or workshop(s)	19 (4.5%)
Other	5 (1.2%)
**Familiarity with AI**	
Not at all familiar	25 (6.0%)
A little familiar	167 (39.8%)
Somewhat familiar	179 (42.6%)
Quite familiar	46 (11.0%)
Extremely familiar	3 (0.7%)
**Use of AI in medical research**	
Yes	37 (8.8%)
No	229 (54.5%)
I do not engage in medical research	154 (36.7%)
**Use of AI in clinical practice**	
Yes	80 (19.0%)
No	340 (81.0%)
**Purpose of AI use in clinical practice** ^a^	
Shared decision-making	17 (4.0%)
Patient monitoring	24 (5.7%)
Outcome prediction	25 (6.0%)
Prediction of complications	9 (2.1%)
Interpretation/quantification of imaging	13 (3.1%)
Grading of disease severity	9 (2.1%)
Diagnosing	40 (9.5%)
Other	19 (4.5%)
**Current role** ^a^	
Owner or employee of practice	319 (76.0%)
Employee of an academic institution	45 (10.7%)
Both Owner or employee of practice and Employee of an academic institution	28 (6.7%)
Other	23 (5.5%)
Decline to state	7 (1.7%)

a. Participants were allowed to select more than one answer for Gender, Training, Type of computer science training/education, Purpose of AI use in clinical practice, and Current role; therefore, total percentages may exceed 100%.

**Table 3:** Coefficients of Models for Primary Outcomes Regressed on Level of Clinical Decision Type, Amount of Information on AI, and Physician to AI

Outcome		Estimated coefficients (p-value) (adjusted p-value^a^)		
		*β*_decision type_	*β*_information_	*β*_exposure_
Confidence	*I am confident in this decision*	−0.045 (0.266) (0.266)	0.144 (0.024) (0.072)	0.042 (0.303) (0.303)
	*The MedAssist result improved the confidence I have in my final decision*	−0.084 (0.225) (0.266)	−0.024 (1.000) (1.000)	**0.274** (0.014) (0.021)
Effectiveness	*Overall, MedAssist improved the care that I was able to provide to the patient*	−0.084 (0.197) (0.266)	0.040 (0.342) (0.513)	**0.350** (0.001) (0.003)

a. *P-values were adjusted using the Benjamini–Hochberg correction (BH procedure) for multiple testing. Adjusted P-values < 0.05 were considered statistically significant; corresponding coefficients are* **bolded**.

## Data Availability

The datasets used and/or analysed during the current study are available from the corresponding author on reasonable request.

## Abbreviations

SMART: Sequential Multiple-Assignment Randomized Trial
AI: Artificial Intelligence
AI-CDSS: AI-enabled Clinical Decision Support System
AI-CDS tools: AI-enabled Clinical Decision Support tools
LLM: Large Language Model
MMS: Medical Marketing Services, Inc
AMA: American Medical Association
